# The Norton Scale at Admission Improves APACHE II-Based Mortality Prediction in the Intensive Care Unit: A Retrospective Cohort Study of 5775 Patients

**DOI:** 10.3390/medicina62071329

**Published:** 2026-07-09

**Authors:** Manuel Tejeda-Adell, José Javier de León-Belmar, Marcelino Pérez-Bermejo, Javier Pérez-Murillo, Sonia Gomar-Vidal, Sara Linares-Aguayo, Antonio Alberto Tortosa-Valencia, Ignacio Ventura, Belén Romero-Gómez, Miriam Martínez-Peris, Alma María Palau-Ferré, María Teresa Murillo-Llorente

**Affiliations:** 1 SONEV Research Group, Faculty of Medicine and Health Sciences, Catholic University of Valencia San Vicente Mártir, C/Quevedo No. 2, 46001 Valencia, Spain; manuel.tejeda@ucv.es (M.T.-A.); deleon_jos@gva.es (J.J.d.L.-B.); javier.perezmu@ucv.es (J.P.-M.); miriam.martinez@ucv.es (M.M.-P.); am.palau@ucv.es (A.M.P.-F.); mt.murillo@ucv.es (M.T.M.-L.); 2 Intensive Care Medicine Service, Manises Health Department, Hospital de Manises, Av. Generalitat Valenciana 50, Manises, 46940 Valencia, Spain; gomar_son@gva.es (S.G.-V.); tortosa_antval@gva.es (A.A.T.-V.); romero_margom1@gva.es (B.R.-G.); 3 Doctoral School, Catholic University of Valencia San Vicente Mártir, C/Quevedo, 2, 46001 Valencia, Spain; 4 Faculty of Medicine and Health Sciences, Catholic University of Valencia San Vicente Mártir, C/Quevedo No. 2, 46001 Valencia, Spain; ignacio.ventura@ucv.es; 5 Data Analysis-BI Unit, Manises Health Department, Hospital de Manises, Av. Generalitat Valenciana 50, Manises, 46940 Valencia, Spain; linares_saragu@gva.es; 6 Translational Research Center San Alberto Magno CITSAM, Catholic University of Valencia San Vicente Mártir, C/Quevedo nº 2, 46001 Valencia, Spain

**Keywords:** Norton scale, APACHE II, intensive care units, hospital mortality, post-ICU mortality, risk assessment, nutritional screening, frailty

## Abstract

*Background and Objectives*: Malnutrition and frailty affect 30–55% of intensive care unit (ICU) patients, yet formal nutritional screening remains inconsistently implemented in routine ICU admission workflows. The APACHE II score, the standard measure of acute physiological severity, does not capture pre-existing nutritional status or functional reserve. The Norton scale, routinely recorded by nursing staff for pressure-ulcer screening, could serve as a pragmatic proxy for the nutritional-functional axis. We assessed its independent prognostic value at admission for in-hospital and post-ICU mortality. *Materials and Methods*: Retrospective cohort study of 5775 consecutive adult patients admitted to a Spanish tertiary polyvalent ICU between 2012 and 2019, with APACHE II and Norton scores recorded at admission. The Norton was analysed as continuous and categorised (minimal >14, medium 13–14, high 10–12, very high 5–9). Discrimination was assessed by AUC and DeLong’s test, predictive improvement by IDI and NRI, and internal validity by bootstrap resampling (B = 200). *Results*: Hospital mortality was 12.8% (*n* = 738), rising from 7.7% in patients with minimal-risk Norton to 34.5% in very high risk. After adjustment for APACHE II, each additional Norton point reduced the odds of death by 7.7% (adjusted OR = 0.923; 95% CI 0.903–0.943). Adding the Norton to APACHE II improved discrimination (AUC 0.865 → 0.872; DeLong *p* = 0.003; IDI = 0.011; continuous NRI = 0.30). In the highest APACHE II quartile, the absolute mortality difference between minimal and very high Norton categories reached 23.2 percentage points. The Norton’s prognostic effect was approximately twice as large for post-ICU mortality (ΔAUC +0.011) as for overall in-hospital mortality (ΔAUC +0.006). *Conclusions*: The Norton scale at admission improves the prognostic capacity of APACHE II in critically ill patients, particularly for post-ICU mortality. Its widespread availability without additional patient-level data collection positions it as a pragmatic candidate for routine prognostic assessment and for guiding targeted nutritional screening.

## 1. Introduction

Risk stratification at admission to the intensive care unit (ICU) remains an essential component of critical care, both to guide individual clinical decisions and to audit quality of care. The Acute Physiology and Chronic Health Evaluation II (APACHE II) score, validated more than four decades ago [1], is the reference standard and combines twelve physiological variables, age, and chronic comorbidities into an aggregated score with good discriminative capacity for hospital mortality [2,3]. However, neither APACHE II nor its updated versions capture nutritional status or pre-admission functional reserve, both recognised as independent predictors of mortality and post-acute complications in multiple cohorts [4,5,6,7,8].

Malnutrition and frailty affect 30–55% of patients admitted to ICUs depending on the diagnostic tool used [4,9], and are consistently associated with increased mortality, longer length of stay, prolonged mechanical ventilation, and worse functional recovery after discharge [4,5,6,7,8]. International guidelines recommend systematic nutritional screening at admission using tools such as the Nutrition Risk in the Critically Ill (NUTRIC) score, the modified NUTRIC (mNUTRIC), the Nutritional Risk Screening 2002 (NRS-2002), or the Global Leadership Initiative on Malnutrition (GLIM) criteria [9,10,11,12,13]. In clinical practice, however, formal nutritional screening at admission is not implemented routinely in a substantial proportion of ICUs across Europe and worldwide, as documented by large international audits such as the nutritionDay surveys [14,15], partly because of nursing time constraints and partly because some components of formal nutritional screening tools (anthropometric measurements, specific biochemical parameters) are not immediately available at admission. There is, therefore, a gap between the evidence supporting nutritional-functional assessment at admission and its effective implementation in routine practice, including in Spanish ICUs, where systematic nutritional screening at admission is likewise not consistently implemented despite the routine availability of nursing-recorded data.

The Norton scale, originally developed in 1962 for pressure-ulcer risk screening [16], is routinely recorded by nursing staff at admission in virtually all Spanish ICUs as part of standard care [17,18], and evaluates five dimensions (general physical condition, mental state, activity, mobility, and incontinence) that partly reflect functional reserve, frailty-related vulnerability [19] and clinical manifestations that may coexist with chronic malnutrition such as sarcopenia, functional decline, and mental deterioration [6,20]. We hypothesised that the Norton scale recorded at admission could act as a pragmatic proxy for the nutritional-functional axis and provide prognostic information independent of APACHE II. To test this hypothesis, we evaluated in a large cohort of consecutive patients admitted to a polyvalent ICU: (1) the association between the Norton score at admission and hospital mortality; (2) the improvement in discriminative capacity when combining the Norton scale and APACHE II; and (3) whether the prognostic effect of the Norton scale is more pronounced for outcomes reflecting cumulative nutritional-functional deterioration (post-ICU mortality, prolonged mechanical ventilation, length of stay) than for outcomes dominated by the acute physiological phase.

## 2. Materials and Methods

### 2.1. Study Design and Population

**Figure 1:**
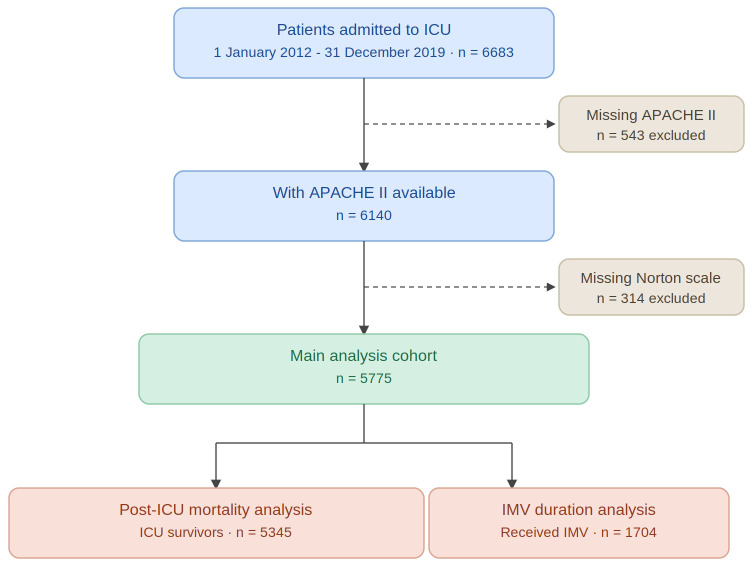
Cohort flow diagram. Of the 6683 patients consecutively admitted to the Intensive Care Unit (ICU) during the study period (1 January 2012–31 December 2019), 543 were excluded for missing APACHE II assessment at admission and 314 for missing Norton assessment at admission. The main analysis cohort included 5775 patients. Post-ICU mortality analyses were restricted to the 5345 survivors of the acute phase; invasive mechanical ventilation (IMV) duration analyses were restricted to the 1704 patients who received it.

Observational, analytical, and retrospective cohort study based on routine clinical data. All adult patients (≥18 years) admitted consecutively to the polyvalent ICU of a Spanish tertiary hospital (a polyvalent ICU is a general, mixed medical-surgical unit that admits undifferentiated critically ill patients across all specialties, rather than a unit dedicated to a single pathology such as cardiac, neurological, or trauma intensive care) during the clinical inclusion period (from 1 January 2012 to 31 December 2019) with admission assessment available for both the APACHE II score and the Norton scale were included. During this period, all variables analysed in the present study were generated and recorded by clinical staff in the electronic health record as part of routine standard care, independently of this research. Retrospective extraction of the anonymised dataset from the electronic health record and all analytical activities for the present study were carried out after the protocol was approved by the Drug Research Ethics Committee (CEIm) of the Hospital Universitario y Politécnico La Fe (code 2021-248-1, approved on 28 April 2021). The inclusion period was deliberately restricted to the interval authorised by the ethics committee and was closed on 31 December 2019 to exclude the SARS-CoV-2 pandemic, which substantially altered the ICU case-mix, admission criteria, and mortality structure; the time elapsed between the end of the inclusion period and publication reflects the completion of the formal ethics approval process and the time required for full anonymised dataset extraction, curation, and analysis. Records with missing data in the main exposure variable (Norton score at admission, *n* = 314), in the main adjustment covariate (APACHE II at admission, *n* = 543), or in the primary outcome were excluded (Figure 1). Of the 6683 patients consecutively admitted during the study period, 908 (13.6%) were excluded for missing values in the exposure or main covariate, leaving 5775 complete cases for the primary analysis. Because both the Norton scale and the APACHE II score are mandatory items in the standardised admission record, missingness was considered most likely to reflect administrative recording gaps rather than a systematic absence of assessment driven by prognosis. However, this assumption cannot be proven retrospectively, and residual selection bias cannot be fully excluded. The large complete-case sample and internal validation with optimism-corrected bootstrap resampling supported the stability of the prognostic models, although bootstrap validation does not eliminate the possibility of bias related to missing data. Reporting follows the STROBE recommendations for observational studies [21] and the TRIPOD statement for prognostic model studies [22].

### 2.2. Variables

The main exposure was the Norton score at admission, calculated by nursing staff following the centre’s standardised procedure and recorded in the electronic health record within the first 24 h. The Norton scale was analysed both as a continuous variable (range 5–20, with lower scores indicating higher risk) and as a categorical variable according to the established cut-off points: minimal risk (>14), medium (13–14), high (10–12), and very high (5–9). The main adjustment covariate was the APACHE II score at admission, recorded by medical staff within the first 24 h. Additional covariates were age and sex. The primary outcome was all-cause in-hospital mortality. Secondary outcomes were as follows: ICU mortality (defined as death during the ICU stay), post-ICU mortality (death after ICU discharge during the same hospital episode, calculated among survivors of the acute phase), ICU length of stay (days), need for invasive mechanical ventilation (IMV), and IMV duration (days) in patients who received it. The end of the critical (acute) phase was operationally defined as the moment of discharge from the ICU, recorded in the electronic health record by the attending intensivist according to standard clinical criteria for resolution of the acute condition (haemodynamic stability, absence of further requirement for organ support, and a clinical decision that continued intensive-level monitoring or treatment was no longer needed). Accordingly, “survivors of the acute phase” were defined as patients alive at the time of ICU discharge, and post-ICU mortality was assessed among this group during the remainder of the same hospital episode.

### 2.3. Statistical Analysis

Continuous variables were described using median and interquartile range (IQR) or mean and standard deviation (SD) depending on their distribution; categorical variables were described using n and percentage. Comparisons between Norton categories were performed using the Kruskal–Wallis test for continuous variables and the χ^2^ test for categorical variables. The choice of non-parametric tests for bivariate comparisons (Kruskal–Wallis) is justified by the ordinal or markedly skewed nature of the variables being compared (the APACHE and Norton clinical scores, length of stay, and duration of mechanical ventilation), distributions for which the median and the interquartile range are more appropriate descriptors than the mean and standard deviation, regardless of sample size. The main analysis of the study (multivariable logistic regression with estimation of AUC, IDI, NRI, and internal validation by bootstrap) is parametric.

**Figure 2:**
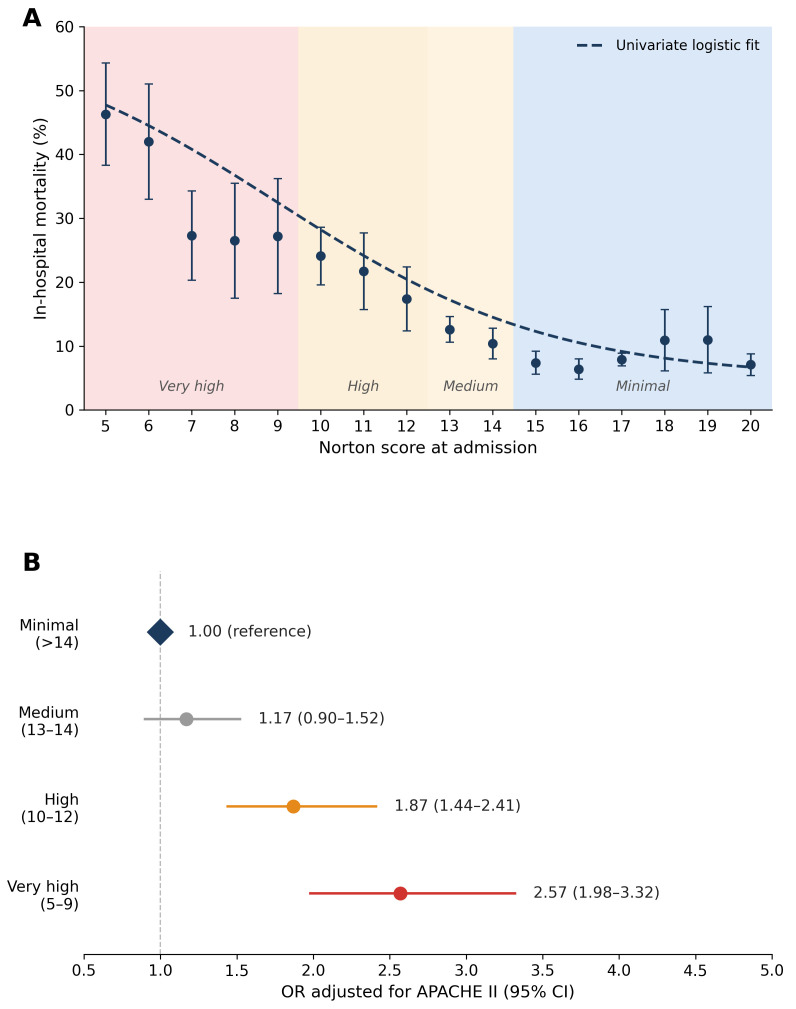
Association between the Norton scale at admission and in-hospital mortality. (**A**) Observed in-hospital mortality (points) by Norton score (5–20), with error bars representing Wilson 95% confidence intervals and a univariate logistic fit line (dashed). The coloured background bands indicate the four risk categories, identified by name below the x-axis. (**B**) Forest plot of the ORs adjusted for APACHE II for each Norton category compared with the reference category (minimal). Points represent the point OR and the lines represent the 95% CI. The vertical dashed line indicates OR = 1. The medium category lost statistical significance after adjustment, whereas the high and very high categories doubled and tripled the risk, respectively.

Five logistic regression models were constructed to predict in-hospital mortality: M0, Norton alone; M1, APACHE II alone; M2, APACHE II + Norton; M3, APACHE II + Norton with an APACHE × Norton interaction term; M4, APACHE II + Norton + age + sex. In these multivariable models the Norton score was entered as a continuous linear term to preserve statistical power and to yield a single, clinically interpretable per-point odds ratio. Given the non-linear dose–response relationship observed in the descriptive analysis (Figure 2A), two complementary strategies were used to ensure that this linear specification did not misrepresent the association or underestimate its predictive contribution: (i) the Norton scale was additionally modelled as a four-level categorical variable using the established cut-off points (Figure 2B); and (ii) a sensitivity analysis was performed in which the continuous Norton term in model M2 was replaced by a restricted cubic spline with knots placed at the 5th, 35th, 65th, and 95th percentiles. The fit of the linear and spline specifications was compared by the likelihood-ratio test and the Akaike Information Criterion, and their discrimination by the AUC. Discriminative capacity was assessed using the area under the ROC curve (AUC) with 95% confidence intervals calculated by the DeLong method, and comparisons between AUCs using the DeLong test for paired samples [23]. The predictive improvement obtained by adding the Norton scale to APACHE II was additionally quantified using the Integrated Discrimination Improvement (IDI) and the Net Reclassification Index (NRI) in its continuous version [24,25]. Calibration was evaluated using calibration plots by deciles of predicted probability and the Hosmer-Lemeshow test; overall goodness of fit was assessed using the Brier score [26]. Internal validity was evaluated by bootstrap resampling with 200 samples and optimism correction using the Harrell method [27].

The simultaneous inclusion of APACHE II and age as covariates in the M4 model introduces a known partial collinearity, since APACHE II contains a categorical age-based scoring component (0–6 points across 5 categories). This decision is maintained for three reasons: (i) the discrete coding of age within APACHE II does not capture the continuous variability of risk within each category; (ii) the original APACHE weights were derived from a 1985 cohort with a different demographic profile, so that the inclusion of age as an independent variable acts as an implicit recalibration; (iii) the variance inflation factors were below 2 for all covariables in the M4 model (APACHE: 1.25; Norton: 1.10; age: 1.15; sex: 1.00), ruling out clinically relevant collinearity. Collinearity was likewise verified for the interaction model (M3): after mean-centring of APACHE II and Norton, the variance inflation factors were 1.16 for APACHE II, 1.14 for Norton, and 1.14 for the APACHE × Norton interaction term, all well below the conventional threshold, confirming that the inclusion of the product term did not introduce relevant collinearity.

Pre-specified analyses of the secondary outcomes: for ICU mortality and post-ICU mortality, logistic models parallel to the main ones were fitted; for post-ICU mortality, the analysis was restricted to survivors of the acute phase (*n* = 5345) to avoid immortal time bias. For ICU length of stay, descriptive statistics were calculated by Norton category and the Kruskal–Wallis test was applied. For IMV, the incidence by Norton category was calculated (χ^2^), and the duration among those who received it (Kruskal–Wallis). All analyses were performed with Python 3.11 (scikit-learn, scipy, and statsmodels packages), and a two-sided *p* value < 0.05 was considered statistically significant. No imputation of missing data was performed; the analysis was restricted to complete cases in the variables of interest.

## 3. Results

### 3.1. Cohort Characteristics

Of the 6683 patients admitted to the ICU during the study period, 5775 met the inclusion criteria (Figure 1). The mean age was 62.7 years (SD 15.4), and 3682 (63.8%) were men. The median APACHE II score was 10 (IQR 6–16) and the median Norton score was 15 (IQR 13–19). Overall, 59.7% of patients were classified as minimal risk according to the Norton scale, 17.7% as medium risk, 12.5% as high risk, and 10.2% as very high risk. Although the coded admission diagnosis was not available, the source of admission allowed a broad case-mix description: 3350 patients (58.0%) were medical or non-surgical admissions, 1915 (33.2%) were scheduled (elective) surgical admissions, and 443 (7.7%) were urgent (emergency) surgical admissions (source of admission was unknown in 67 patients, 1.2%). In-hospital mortality differed markedly across these groups, being lowest in scheduled surgical patients (4.5%) and highest in urgent surgical (20.3%) and medical (16.5%) admissions, consistent with the expected severity profile of each pathway. Overall in-hospital mortality was 12.8% (*n* = 738). Baseline characteristics by Norton category are shown in Table 1, with a significant gradient (*p* < 0.001 for all comparisons) observed across all main variables: lower Norton scores were associated with older age, higher APACHE II scores, higher mortality, and greater use of life-supporting interventions.

### 3.2. Norton Score at Admission and In-Hospital Mortality

In-hospital mortality increased monotonically from 7.7% (265/3446) in patients with minimal-risk Norton scores to 34.5% (203/588) in those with very high risk (*p* < 0.001). In the univariate model (M0), each additional Norton point was associated with a 15.0% reduction in the odds of in-hospital mortality (crude OR = 0.848; 95% CI 0.832–0.864; *p* < 0.001), with an AUC of 0.675 (95% CI 0.652–0.692). The dose–response relationship was non-linear: risk increased markedly below a Norton score of 14 and plateaued above it (Figure 2A).

### 3.3. Independent Prognostic Value and Discriminative Improvement

After adjustment for APACHE II (M2), each additional Norton point remained independently associated with lower mortality (adjusted OR = 0.923; 95% CI 0.903–0.943; *p* < 0.001), approximately half of the univariate effect, indicating that part of the crude effect is explained by the correlation with the physiological severity captured by APACHE II while the other half is independent information. The full coefficients of the five models are presented in Table 2. In the categorical analysis adjusted for APACHE II, compared with the reference category (minimal risk), the medium category lost statistical significance (OR = 1.17; 95% CI 0.90–1.52; *p* = 0.236), whereas the high category (OR = 1.87; 95% CI 1.44–2.41; *p* < 0.001) and the very high category (OR = 2.57; 95% CI 1.98–3.32; *p* < 0.001) doubled and tripled the risk, respectively (Figure 2B). This pattern is consistent with the non-linearity observed in Figure 2A: the prognostic information additional to APACHE II derives mainly from patients with a Norton score ≤ 12.

At equal physiological severity, patients with worse Norton scores had absolutely higher mortality across all APACHE II quartiles, with a gradient that became more pronounced in the upper quartiles. In the most severe quartile (APACHE II ≥ 16), mortality was 30.4% in patients with minimal Norton risk compared with 53.6% in those with very high Norton risk, an absolute difference of 23.2 percentage points (*p* < 0.001).

**Figure 3:**
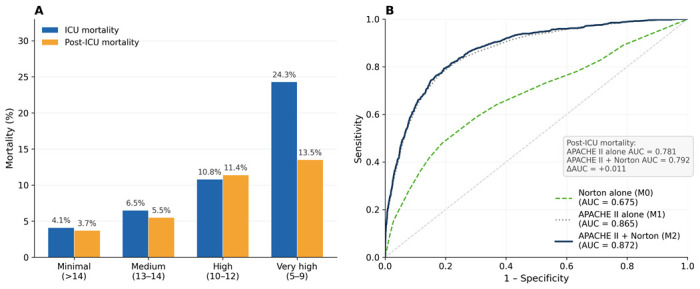
Comparison of prognostic capacity for ICU mortality versus post-ICU mortality. (**A**) ICU mortality (dark blue bars) and post-ICU mortality among survivors of the acute phase (orange bars) by Norton category at admission. The slope of the Norton-mortality gradient is proportionally larger in the post-ICU phase, consistent with the interpretation of the Norton scale as a nutritional-functional proxy. (**B**) ROC curves for the prediction of in-hospital mortality of models M0 (Norton alone, light green line), M1 (APACHE II alone, dashed grey line) and M2 (APACHE II + Norton, thick navy line). Adding the Norton scale to APACHE II significantly improves discrimination (DeLong test *p* = 0.003); the improvement is approximately twice as large when the outcome is post-ICU mortality (ΔAUC = +0.011) as when it is overall in-hospital mortality (ΔAUC = +0.006).

**Figure 4:**
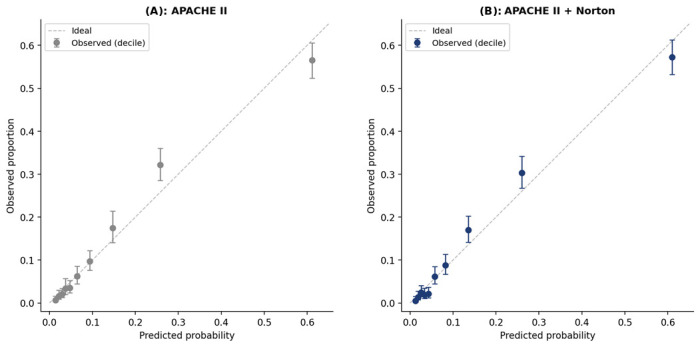
Calibration plots by deciles of predicted probability for the prediction of in-hospital mortality. (**A**) APACHE II model (M1). (**B**) APACHE II + Norton model (M2). For each decile of predicted risk, the observed proportion of deaths (points, with Wilson 95% confidence intervals) is plotted against the mean predicted probability; the dashed diagonal line represents perfect calibration. Both models show close agreement between predicted and observed mortality across the whole range of risk, indicating that the statistically significant Hosmer-Lemeshow test (*p* < 0.001) reflects the sensitivity of that test to the large sample size rather than a clinically relevant miscalibration.

Adding the Norton scale to APACHE II significantly improved discrimination (AUC: 0.865 → 0.872; DeLong test *p* = 0.003), with complementary indices IDI = 0.011 (95% CI 0.007–0.015) and continuous NRI = 0.30 (Table 3; the ROC curves are shown in Figure 3B). The decomposition of the NRI showed that the improvement was mainly driven by better reclassification of non-events (non-event NRI = 0.19) than of events (event NRI = 0.10). The subsequent addition of age and sex (M4) raised the AUC to 0.875 (DeLong *p* < 0.001). The Hosmer-Lemeshow test was statistically significant in all models (χ^2^ = 34.7–42.8; *p* < 0.001), an expected finding in large samples and attributable to small absolute discrepancies in individual deciles; the calibration plots by deciles of predicted probability (Figure 4) showed close agreement between predicted and observed mortality across the whole range of risk for both M1 and M2, confirming that the significant Hosmer-Lemeshow statistic reflects the sensitivity of the test to the large sample size rather than a clinically relevant miscalibration. The Brier score, more robust to sample size, showed a consistent improvement (M1: 0.081; M2: 0.079; M4: 0.079). The bootstrap optimism was below 0.001 in all models, indicating absence of material overfitting. In the sensitivity analysis modelling the Norton scale with a restricted cubic spline instead of a linear term, the discrimination of model M2 was essentially unchanged (AUC 0.872 for both specifications), the likelihood-ratio test did not show a significant advantage of the spline over the linear specification (χ^2^ = 5.72; df = 2; *p* = 0.057), and the Akaike Information Criterion was virtually identical (linear 3070.7; spline 3069.0). The linear specification was therefore retained for the main models because the spline did not materially improve discrimination or overall model fit, while the linear term preserves interpretability; the categorical analysis (Figure 2B) is provided alongside it to display the non-linear pattern in clinically meaningful strata.

### 3.4. Pre/Post-ICU Asymmetry

To assess whether the prognostic effect of the Norton scale is more pronounced for outcomes sensitive to nutritional-functional deterioration, we separated in-hospital mortality into ICU mortality (during the critical pathophysiological phase) and post-ICU mortality (among survivors of the critical phase, *n* = 5345). In the ICU, mortality increased from 4.1% (minimal) to 24.3% (very high). After ICU discharge, it increased from 3.7% to 13.5% (a proportionally larger gradient in relative terms) (Figure 3A). More importantly, the AUC improvement obtained by adding the Norton scale to APACHE II was approximately twice as large for post-ICU mortality (ΔAUC = +0.011; APACHE II alone 0.781 → APACHE II + Norton 0.792) as for overall in-hospital mortality (ΔAUC = +0.006; Figure 3B). The likelihood ratio test for adding the Norton scale to the APACHE II model to predict post-ICU mortality was highly significant (χ^2^ = 31.26; df = 1; *p* < 0.001). The adjusted β coefficient for the Norton scale was −0.085 for post-ICU mortality compared with −0.078 for overall in-hospital mortality.

### 3.5. Secondary Outcomes

The mean ICU length of stay increased from 2.8 days (median 2; IQR 1–3) in patients with minimal Norton risk to 6.8 days (median 3; IQR 2–7) in those with very high Norton risk (*p* < 0.001). The proportion of patients who required IMV increased from 24.7% to 69.0% across the same categories (χ^2^ = 632.4; *p* < 0.001), although the relationship was not strictly monotonic between the minimal and medium categories. Among those who received IMV, duration was substantially prolonged: the 75th percentile rose from 2 days in patients with minimal Norton risk to 7 days in those with very high risk. The complete data for secondary outcomes are presented in Table 4.

## 4. Discussion

In this cohort of 5775 patients consecutively admitted to a polyvalent ICU, the Norton scale at admission provided prognostic information independent of APACHE II for in-hospital mortality, with a statistically significant but clinically modest improvement in overall discrimination (ΔAUC = +0.006). We emphasise that this gain should be interpreted as incremental rather than transformative: APACHE II already discriminates well, and the added value of the Norton scale lies not in a large increase in global discrimination but in the fact that this incremental, potentially clinically useful information is obtained from data already collected at the bedside, with no additional patient-level measurement. The most clinically relevant finding is the asymmetry observed between the mortality components: the prognostic effect of the Norton scale was approximately twice as large for post-ICU mortality (ΔAUC = +0.011) as for overall in-hospital mortality (ΔAUC = +0.006). This asymmetry is consistent with the interpretation of the Norton scale as a proxy for the pre-existing nutritional-functional axis: mortality in the acute phase is dominated by the pathophysiological severity that APACHE II captures adequately, whereas mortality after ICU discharge appears to reflect, to a greater extent, the patient’s limited functional reserve to overcome the post-critical phase in a context of accumulated frailty and possible malnutrition [6,7,8], dimensions that APACHE II does not assess specifically.

The nutritional-functional interpretation of the Norton scale has a plausible pathophysiological basis, although it should be understood as indirect. The five dimensions of the scale (general physical condition, mental state, activity, mobility, and incontinence) do not correspond directly to formal nutritional diagnostic criteria such as GLIM, which require evidence of phenotypic and etiologic components including weight loss, low body mass index, reduced muscle mass, reduced intake or assimilation, and inflammation [11]. However, low Norton scores may capture downstream functional manifestations that frequently coexist with frailty and chronic malnutrition, including sarcopenia, reduced mobility, dependence, and impaired general condition [6,19,28]. The scale was not designed to assess nutritional status, but these functional dimensions may identify a vulnerable subgroup with limited physiological reserve. This interpretation is reinforced by the non-linear pattern observed: the prognostic information additional to APACHE II comes mainly from patients with a Norton score ≤ 12, a range in which frailty, dependency, and clinically relevant nutritional vulnerability are more likely to coexist. Importantly, the present study was not designed to validate the Norton scale as a nutritional assessment tool, but to evaluate whether this routinely recorded nursing scale provides incremental prognostic information on mortality in critically ill patients.

Our results are consistent with previous literature. Independent studies have shown that frailty as measured by the Clinical Frailty Scale is associated with in-hospital mortality and post-ICU mortality with effects of similar magnitude [4,5,29]; that nutritional screening tools such as NUTRIC and mNUTRIC improve the discrimination of APACHE II in selected cohorts [12,13]; and that sarcopenia assessed by muscle ultrasound is associated with post-ICU mortality and functional dependence [6]. Previous specific studies on the prognostic value of the modified Norton scale in critically ill patients have pointed in the same direction, although with smaller cohorts [20,28]. Our findings should also be framed within the broader concept of repurposing routinely recorded nursing or clinical scales, originally designed for other purposes, to inform prognosis without requiring additional patient-level measurements. The Norton scale is one such instrument, but it is not the only possible candidate. The Braden scale, also widely used for pressure-ulcer risk assessment, captures related domains such as mobility, activity, nutrition, and sensory perception, and could plausibly provide comparable prognostic information. Similarly, structured frailty instruments may offer more specific information where they are routinely collected. In the present cohort, however, neither the Braden scale nor a formal frailty score was consistently available, preventing a direct head-to-head comparison. The choice of the Norton scale was therefore not based on an assumption of superiority over other pressure-ulcer or frailty-related instruments, but on its institutional implementation and systematic availability in the electronic admission record during the study period. Future prospective studies should therefore compare the Norton scale with other routinely recorded nursing scales and with formal nutritional and frailty instruments measured in the same patients. Formal nutritional screening tools provide more specific information, but their integration into admission workflows may be limited by the need for additional clinical, anthropometric, nutritional, or laboratory data, depending on the tool used and the local setting. The relevant clinical question is therefore not “Norton versus NUTRIC”, but whether a routinely available Norton score could help identify patients who should subsequently undergo formal nutritional assessment and targeted post-ICU follow-up.

This study has several limitations that should be made explicit. First, it is a single-centre, retrospective study based on routine clinical data; generalisation to other centres with a different case-mix requires external validation. Second, we cannot guarantee uniform quality of Norton scale recording or control for variations in its assessment between professionals, although its simplicity and prolonged use by nursing staff minimise this risk. Third, a total of 908 patients (13.6% of those admitted during the study period) were excluded because of missing Norton or APACHE II values at admission and were therefore not included in the complete-case analysis. Because both scores are mandatory items of the standardised admission record, this missingness was considered more likely to reflect administrative recording gaps than a systematic absence of assessment driven by prognosis. Nevertheless, we cannot demonstrate this assumption retrospectively, and residual selection bias cannot be excluded. The large analysed cohort and the internal bootstrap validation support model stability, but they do not fully rule out bias related to missing data. Fourth, we do not have specific nutritional variables (NUTRIC, albumin, prealbumin, muscle mass by ultrasound) that would allow direct quantification of the fraction of the Norton scale’s effect mediated by malnutrition; the nutritional interpretation, although pathophysiologically plausible, remains indirect. In particular, formal nutritional screening (NUTRIC, mNUTRIC, or GLIM) was not systematically recorded during the study period, so we are unable to report how the Norton scale performs relative to a formal nutritional score in the same patients; this direct comparison is a priority for prospective validation. Fifth, we do not have the coded ICD admission diagnosis, which prevents adjustment for disease category (medical/surgical, sepsis/trauma/respiratory). Although APACHE II captures much of the acute physiological severity at admission, residual disease-category effects could persist despite the broad admission-pathway breakdown provided. Sixth, the primary outcome is in-hospital mortality; we do not have 30-day, 90-day, or one-year mortality, nor quality of life or functional status after discharge, outcomes that would be particularly relevant for assessing the functional component of the Norton scale. Seventh, validation is internal by bootstrap; no external validation has been performed in independent cohorts, an essential step before any formal proposal of a recalibrated “APACHE-Norton”.

The most immediate clinical implication of our findings is that the Norton scale may be considered as a complementary element in routine prognostic assessment at ICU admission, alongside APACHE II. Because it is already recorded by nursing staff as part of pressure-ulcer risk screening, its use for risk stratification would not require additional patient-level data collection. However, its formal adoption as a prognostic tool would require protocol updating, staff training, standardisation of recording, and quality-control audits. The finding that the prognostic value of the Norton scale is particularly pronounced for mortality after ICU discharge also suggests a reasonable hypothesis for future research: admission Norton scores could be used to prioritise patients for formal nutritional screening and structured post-ICU follow-up. Prospective validation of this stepwise strategy, ideally in a multicentre pragmatic trial, remains a research priority.

## 5. Conclusions

The Norton scale at admission, routinely recorded by nursing staff as part of pressure-ulcer risk assessment and requiring no additional patient-level data collection, provides prognostic information beyond APACHE II in critically ill patients, with a particularly relevant association with mortality after ICU discharge. Its incorporation into routine prognostic assessment appears feasible, although its programmatic use as a prognostic tool would require protocol updating, staff training, standardisation of recording, and quality-control auditing. The translation of this prognostic value into targeted nutritional, rehabilitation, or post-ICU follow-up interventions requires prospective validation. Future research should externally validate the incremental prognostic value of the admission Norton scale in independent multicentre cohorts, extend the outcome horizon to 30-day, 90-day, and one-year mortality and post-discharge functional status, assess its prognostic performance across clinically relevant ICU subgroups such as respiratory, cardiac, septic, surgical, and medical patients, compare Norton head-to-head with Braden and formal nutritional and frailty instruments, and test whether a stepwise strategy triggered by low Norton scores improves clinical outcomes and is cost-effective.

## Figures and Tables

**Table 1:** Baseline characteristics of the cohort by Norton category at admission.

Characteristic	Total *n* = 5775	Minimal (>14) *n* = 3446	Medium (13–14) *n* = 1022	High (10–12) *n* = 719	Very High (5–9) *n* = 588
Age, mean (SD), years	62.7 (15.4)	61.5 (15.3)	64.6 (15.2)	65.2 (15.3)	63.7 (15.3)
Men, n (%)	3682 (63.8)	2258 (65.5)	637 (62.3)	421 (58.6)	366 (62.2)
APACHE II, median (IQR)	10 (6–16)	9 (5–13)	10 (7–17)	13 (8–20)	17 (10–25)
IMV during admission, n (%)	1704 (29.5)	850 (24.7)	219 (21.4)	229 (31.8)	406 (69.0)
IMV duration, median (IQR), d ^2^	1 (1–2)	1 (1–2)	1 (1–3)	2 (1–7)	2 (1–7)
IMV duration, mean (SD), d ^2^	3.0 (5.9)	3.0 (5.9)	3.7 (6.5)	6.1 (9.0)	6.4 (9.6)
ICU LOS, median (IQR), d	2 (1–3)	2 (1–3)	2 (1–3)	2 (1–5)	3 (2–7)
ICU LOS, mean (SD), d	3.4 (5.6)	2.8 (4.2)	3.0 (4.0)	4.5 (6.9)	6.8 (10.1)
Hospital LOS, median (IQR), d	8 (5–15)	8 (5–14)	8 (5–14)	9 (6–17)	10 (5–19)
In-hospital mortality, n (%)	738 (12.8)	265 (7.7)	119 (11.6)	151 (21.0)	203 (34.5)
ICU mortality, n (%)	430 (7.4)	143 (4.1)	66 (6.5)	78 (10.8)	143 (24.3)
Post-ICU mortality, n (%) ^1^	308 (5.8)	122 (3.7)	53 (5.5)	73 (11.4)	60 (13.5)

^1^ Calculated among survivors of the acute phase (*n* = 5345). ^2^ Calculated among patients who received invasive mechanical ventilation (*n* = 1704). IQR, interquartile range; SD, standard deviation; IMV, invasive mechanical ventilation; LOS, length of stay. Differences between categories were significant at *p* < 0.001 for all variables (Kruskal–Wallis or χ^2^ test).

**Table 2:** Logistic regression models for in-hospital mortality.

Model/Variable	β (SE)	OR (95% CI)	*p*	AIC/AUC
M0—Norton alone				4122.3/0.675
Norton	−0.165 (0.010)	0.848 (0.832–0.864)	<0.001	
M1—APACHE II alone				3119.8/0.865
APACHE II	+0.166 (0.006)	1.181 (1.168–1.194)	<0.001	
M2—APACHE II + Norton				3070.7/0.872
APACHE II	+0.157 (0.006)	1.170 (1.157–1.183)	<0.001	
Norton	−0.080 (0.011)	0.923 (0.903–0.943)	<0.001	
M3—APACHE II × Norton				3063.7/0.871
APACHE II (centred)	+0.163 (0.006)	1.177 (1.163–1.191)	<0.001	
Norton (centred)	−0.109 (0.015)	0.896 (0.871–0.923)	<0.001	
APACHE II × Norton	+0.0039 (0.0013)	1.004 (1.001–1.006)	0.002	
M4—APACHE II + Norton + age + sex				3049.1/0.875
APACHE II	+0.152 (0.006)	1.164 (1.151–1.178)	<0.001	
Norton	−0.083 (0.011)	0.920 (0.900–0.941)	<0.001	
Age	+0.018 (0.004)	1.018 (1.011–1.026)	<0.001	
Male sex	−0.045 (0.098)	0.956 (0.789–1.158)	0.647	

β: regression coefficient; SE: standard error; OR: odds ratio; CI: confidence interval; AIC: Akaike Information Criterion; AUC: area under the ROC curve.

**Table 3:** Discriminative capacity and prognostic improvement indices for in-hospital mortality.

Model	AUC (95% CI)	ΔAUC vs. APACHE II	*p* (DeLong)
Norton alone (M0)	0.675 (0.653–0.699)	−0.190	<0.001
APACHE II alone (M1)	0.865 (0.851–0.879)	Reference	–
APACHE II + Norton (M2)	0.872 (0.858–0.885)	+0.006	0.003
APACHE II + Norton + age + sex (M4)	0.875 (0.861–0.888)	+0.010	<0.001

Complementary indices for adding the Norton scale to APACHE II (M2 vs. M1): IDI = 0.011 (95% CI 0.007–0.015); continuous NRI = 0.30 (NRI events 0.10; NRI non-events 0.19). Brier scores: M1 = 0.081; M2 = 0.079; M4 = 0.079. Bootstrap optimism (B = 200): <0.001 for all models. AUC, area under the ROC curve; CI, confidence interval; IDI, Integrated Discrimination Improvement; NRI, Net Reclassification Index.

**Table 4:** Secondary outcomes by Norton category at admission.

Outcome	Minimal (>14)	Medium (13–14)	High (10–12)	Very High (5–9)
ICU mortality, %	4.1	6.5	10.8	24.3
Post-ICU mortality, % ^1^	3.7	5.5	11.4	13.5
ICU LOS, mean (d)	2.8	3.0	4.5	6.8
ICU LOS, median (IQR)	2 (1–3)	2 (1–3)	2 (1–5)	3 (2–7)
Need for IMV, %	24.7	21.4	31.8	69.0
IMV duration, median (d) ^2^	1	1	2	2
IMV duration, 75th percentile (d) ^2^	2	3	7	7

^1^ Calculated among survivors of the acute phase (*n* = 5345). ^2^ Calculated among patients who received IMV (*n* = 1704). *p* < 0.001 for all comparisons. ICU, intensive care unit; IMV, invasive mechanical ventilation; LOS, length of stay; IQR, interquartile range.

## Data Availability

The raw data supporting the conclusions of this article will be made available by the authors on request.

## Abbreviations

The following abbreviations are used in this manuscript:

AIC	Akaike Information Criterion
APACHE II	Acute Physiology and Chronic Health Evaluation II
ASPEN	American Society for Parenteral and Enteral Nutrition
AUC	Area under the receiver operating characteristic curve
CEIm	Comité de Ética de la Investigación con Medicamentos (Drug Research Ethics Committee)
CRediT	Contributor Roles Taxonomy
DeLong test	Statistical test for comparing areas under correlated ROC curves
ESPEN	European Society for Clinical Nutrition and Metabolism
GDPR	General Data Protection Regulation
GLIM	Global Leadership Initiative on Malnutrition
ICD	International Classification of Diseases
ICU	Intensive care unit
IDI	Integrated Discrimination Improvement
IMV	Invasive mechanical ventilation
IQR	Interquartile range
LOS	Length of stay
mNUTRIC	Modified Nutrition Risk in the Critically Ill score
NRI	Net Reclassification Index
NRS-2002	Nutritional Risk Screening 2002
NUTRIC	Nutrition Risk in the Critically Ill score
ROC	Receiver operating characteristic
STROBE	Strengthening the Reporting of Observational Studies in Epidemiology
TRIPOD	Transparent Reporting of a multivariable prediction model for Individual Prognosis Or Diagnosis
